# Combination treatment with decitabine and ionizing radiation enhances tumor cells susceptibility of T cells

**DOI:** 10.1038/srep32470

**Published:** 2016-09-27

**Authors:** Cheol-Hun Son, Hong-Rae Lee, Eun-Kyoung Koh, Dong-Yeok Shin, Jae-Ho Bae, Kwangmo Yang, You-Soo Park

**Affiliations:** 1 Department of Research Center, Dongnam Institute of Radiological & Medical Sciences, Jwadong-gil 40, Jangan-eup, Gijang-gun, Busan, 46033, Republic of Korea; 2 Department of Biochemistry, Pusan National University School of Medicine, Yangsan, 50612, Republic of Korea

## Abstract

Decitabine has been found to have anti-metabolic and anti-tumor activities in various tumor cells. Recently, the use of decitabine in combination with other conventional therapies reportedly resulted in improved anti-tumor activity against various tumors. Ionizing radiation (IR) is widely used as a cancer treatment. Decitabine and IR improve immunogenicity and susceptibility of tumor cells to immune cells by up-regulating the expression of various molecules such as major histocompatibility complex (MHC) class I; natural-killer group 2, member D (NKG2D) ligands; and co-stimulatory molecules. However, the effects of combining decitabine and IR therapies are largely unknown. Our results indicate that decitabine or IR treatment upregulates MHC class I, along with various co-stimulatory molecules in target tumor cells. Furthermore, decitabine and IR combination treatment further upregulates MHC class I, along with the co-stimulatory molecules, when compared to the effect of each treatment alone. Importantly, decitabine treatment further enhanced T cell-mediated cytotoxicity and release of IFN- γ against target tumor cells which is induced by IR. Interestingly, decitabine did not affect NKG2D ligand expression or NK cell-mediated cytotoxicity in target tumor cells. These observations suggest that decitabine may be used as a useful immunomodulator to sensitize tumor cells in combination with other tumor therapies.

The cytosine analog, 5-aza-2-deoxycytidine (decitabine), is a drug that induces epigenetic effects without altering the DNA sequence via DNA hypomethylation. Decitabine, a DNA methyltransferase (DNMTs) inhibitor^1^, directly inhibits tumor growth and enhances the therapeutic effects of drugs administered concomitantly by increasing the expression of tumor suppressor genes^2^,^3^ including those encoding for major histocompatibility complex (MHC) class I^4^,^5^,^6^,^7^. In addition, decitabine increases the expression of tumor antigens by inducing epigenetic remodeling, thereby improving tumor immunogenicity^5^,^8^. Thus, decitabine may be used as an adjuvant agent for cancer immunotherapy and an anti-tumor drug^2^,^5^. It has shown significant anti-tumor effects in patients with hematopoietic malignancies and myelodysplastic syndrome (MDS), but not in patients with solid tumors^9^.

Recently, combining immunotherapy with decitabine has been reported to further enhance the anti-tumor effects of immunotherapy in solid tumors^10^. Decitabine increases macrophage cytotoxicity, dendritic cell (DC) activation, and macrophage M1 polarization, while reducing CD11b^+^Gr1^+^ myeloid-derived suppressor cells (MDSC)^11^. It is also known to improve tumor cell susceptibility to NK cell-mediated lysis by increasing NKG2D ligand expression^12^,^13^,^14^. NKG2D is an important immunoreceptor that induces NK cell activation. NKG2D ligands such as MHC class I-related chain A and B (MICA/B) and UL-16 binding proteins (ULBPs) are upregulated by various stressors, including heat shock, ionizing radiation, anti-tumor drugs, oxidative stress, and viral infections; they also show various expression patterns in different cancer cells^15^,^16^,^17^,^18^. However, tumor cells have the capacity to downregulate NKG2D ligand expression and escape immune recognition. Previous studies have reported that NKG2D ligand methylation contributes to immune system evasion of tumor cells, whereas decitabine increases NKG2D ligand expression in tumor cells^19^.

Ionizing radiation (IR) is widely used as a treatment in cancer patients; it causes double-strand DNA breaks, and thus, induces cancer cell death. IR-induced cancer cell death provides a good source of antigens for DC uptake and presentation to T cells^20^,^21^. Furthermore, IR upregulates immune stimulatory receptors such as Fas/CD95 and MHC class I, and co-stimulatory molecules. It also induces the proinflammatory cytokines interleukin (IL)-1β and tumor necrosis factor (TNF)-α^22^,^23^,^24^,^25^.

The immune system uses human leukocyte antigens (HLAs) to distinguish between self and non-self cells. For proper immune system functioning, NK cell activity is regulated through a balance of activating and inhibitory signals. Furthermore, MHC class I-deficient tumors or infected cells are highly sensitive to NK cells^26^. Recent studies have shown that the expression of HLA-B is inhibited in esophageal squamous cell carcinomas (ESCC) by hypermethylation, a phenomenon that was reversed by treatment with decitabine^7^. Furthermore, decitabine treatment of melanoma cells resulted in increased HLA-A and HLA-B expression^4^,^6^. In addition, when NK cells are directly exposed to decitabine, an increase in the expression of killer cell immunoglobulin-like receptor (KIR) is observed, along with reduced NK cell cytolytic activity^27^. In contrast to NK cells, the T cell response to decitabine is mediated through the T cell receptor (TCR), which interacts with MHC molecules on target cell membranes. Additionally, co-stimulatory molecules expressed on target cell membranes further increase the T cell response. Previous studies reported that decitabine and IR upregulate the expression of MHC and co-stimulatory molecules on tumor cells, resulting in an efficient anti-tumor T cell response^28^,^29^,^30^,^31^.

While previous studies have focused on the effects of decitabine or IR treatment alone, their combined effects on the activity of immune cells have not been reported. In this study, we investigated whether decitabine and IR combination treatment enhances tumor cell susceptibility to immune cells, with a focus on T cells.

## Results

### Decitabine’s effects on tumor cell viability

**Figure 1:**
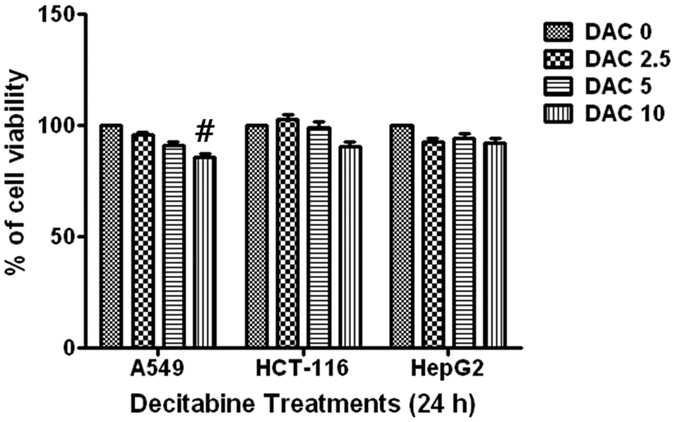
Effects of decitabine on tumor cell viability. Cells were seeded at an initial density of 1 × 10^5 ^cells/well in 6-well tissue culture plates and incubated for 24 h then treated with various concentrations of decitabine for 24 h. Cell viability was evaluated using an MTT assay. Results are expressed as percentage of the vehicle treated control ± standard deviation (SD) of three separate experiments. Statistical significance was determined using a Student’s t-test. ^#^*P* < 0.05 (^#^untreated control *versus* decitabine).

To determine decitabine’s effects on tumor cell viability, an MTT assay was performed 24 h after decitabine treatment (0–10 μM). Treatment with decitabine at a concentration of 0–5 μM caused no cytotoxicity in tumor cell lines; however, the highest decitabine concentration tested (10 μM) resulted in approximately 20% inhibition of cell growth compared with that in controls in A549 cells (Fig. 1). Therefore, we used decitabine at a concentration of 5 μM for all remaining experiments, including studies of immunogenicity and tumor susceptibility to NK and T cells in various tumor cell lines.

### Effects of decitabine and IR combination treatment on MHC class I and co-stimulatory molecules expression

**Figure 2:**
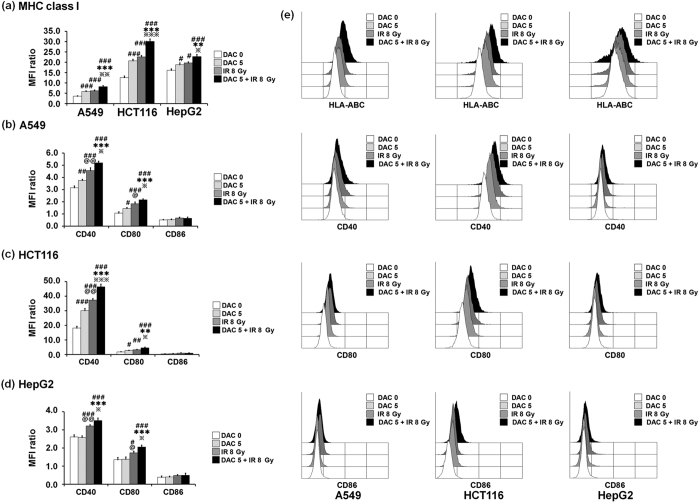
Expression of MHC class I and co-stimulatory molecules in tumor cells after treatment with decitabine, ionizing radiation (IR), or their combination. Tumor cells were treated with 5 μM decitabine for 24 h then exposed to radiation doses of 8 Gy. After 24 h, cells were harvested and the expression of MHC class 1 molecule in various tumor cells (**a**) and the expression of co-stimulatory molecules in A549 cells (**b**), HCT-116 cells (**c**), and HepG2 cells (**d**) were analyzed by flow cytometry. Mean fluorescence intensity (MFI) ratio was calculated as MFI with specific mAb/MFI with isotype control mAb (**a**–**d**). In the figure, filled white represents the untreated control, filled light gray represents decitabine, filled dark gray represents IR and filled black represents decitabine and IR combination treatment (**e**). Results are expressed as the average MFI ± SD. Experiments were independently performed from five healthy donors. The assay was performed in triplicated each donor. Statistical significance was determined using a one-way ANOVA. ^#^*P* < 0.05, ^##^*P* < 0.005, ^###^*P* < 0.0005 (^#^DAC 0 *versus* other groups). **P < 0.005, ***P < 0.0005 (*DAC 5 *versus* other groups). ^**^P < 0.05, ^******^P < 0.0005 (^**^IR *versus* DAC 5 + IR 8 Gy). ^@^*P* < 0.05, ^@@^*P* < 0.005, ^@@@^*P* < 0.0005 (^@^DAC 5 *versus* IR 8 Gy).

IR and epigenetic modulating agents are known to enhance the expression of several genes in tumor cells, including MHC class I and its co-stimulatory molecules^28^,^29^,^30^,^31^,^32^,^33^. Therefore, we investigated whether treatment with decitabine and IR alone or in combination up-regulates MHC class I expression and expression of co-stimulatory molecules in tumor cells. A549, HCT-116, and HepG2 cells were treated with 5 μM decitabine for 24 h. Then, the tumor cells were exposed to radiation doses of 8 Gy. After 24 h, the surface expression of MHC class I, along with co-stimulatory molecules, on tumor cells was analyzed using flow cytometry. As shown in Fig. 2, MHC class I molecules were significantly increased in all target tumor cells treated with decitabine or IR compared with untreated control cells. Furthermore, the combination treatment further significantly increased the expression of MHC class I molecules compared with either treatment alone. In addition, treatment with decitabine or IR significantly increased the expression of co-stimulatory molecules such as CD40 and CD80 in A549 and HCT-116 cells. However, these co-stimulatory molecules were not increased in HepG2 cells treated with decitabine alone. Importantly, the combination treatment further significantly increased the expression of these co-stimulatory molecules compared with either treatment alone. On the other hand, tumor cells treated with decitabine, IR, or their combination did not show changes in CD86 expression levels. These results suggest that treatment with decitabine and/or IR significantly increased the expression of MHC class I and co-stimulatory molecules such as CD40 and CD80. Therefore, the combination treatment may further enhance immunogenicity of tumor cells; however, the effect is dependent on cell type.

### Effects of decitabine and IR combination treatment on T cell activation

**Figure 3:**
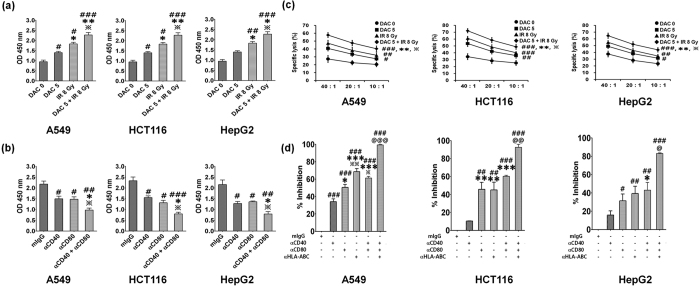
Effects of treatment with decitabine, ionizing radiation (IR), or their combination on activity of T cells against target tumor cells. T cells were co-cultured with decitabine, IR or both treated tumor cells (A549, HCT-116, and HepG2 cells) for 5days for MLR assay. Also, T cells were co-cultured with decitabine, IR or both treated tumor cells at various ratios for 4 h for cytotoxicity assay. For blockade experiments, T cells were co-cultured with combination-treated target tumor cells in the presence of 10 ug/ml anti-CD40, CD80, and/or HLA-A,B,C antibody. T cell proliferation (**a**) or inhibition (**b**) was assessed using Cell Counting Kit-8 (CCK8), and T cell cytotoxicity (**c**) or inhibition of cytotoxicity (**d**) was performed by flow cytometry. Percent inhibition of cytotoxicity was calculated as a percentage of the inhibition by the isotype control antibody. Results express the average T cell proliferation or T cell-mediated cytotoxicity ± SD in A549, HCT-116, and HepG2 cells. Experiments were independently performed from five healthy donors. The assay was performed in triplicated each donor. The statistical significance was determined using a one-way ANOVA. ^#^*P* < 0.05, ^##^*P* < 0.005, ^###^*P* < 0.0005 (^#^DAC 0 *versus* other groups, mIgG *versus* other groups). *P < 0.05, **P < 0.005, ***P < 0.005 (*DAC 5 *versus* other groups, αCD40 *versus* other groups). ^**^P < 0.05, *P* < 0.005, *P* < 0.0005 (^**^IR 8 Gy *versus* DAC 5 + IR 8 Gy, αCD80 *versus* αCD40 + αCD80). ^@^*P* < 0.05, ^@@^*P* < 0.005 (^@^αCD40 + αCD80 *versus* αCD40 + αCD80 + αHLA-ABC).

Decitabine and IR combination treatment significantly increased MHC class I and co-stimulatory molecules in tumor cells (Fig. 2). To investigate role of treatment with decitabine and/or IR-induced co-stimulatory molecules expression in stimulation of T cells, we performed mixed lymphocyte culture using the decitabine, IR or both treated tumor cells and co-stimulatory molecules (CD40 and CD80) blockade experiments using the combination-treated tumor cells. Decitabine or IR-treated tumor cells induced stronger T cell proliferation compared with untreated tumor cells (Fig. 3a). However, decitabine-treated HepG2 cells induced very weak T cell proliferation. In particular, the combination treatment further significantly increased T cell proliferation compared with either treatment alone. In contrast, blockade of co-stimulatory molecules (CD40, CD80 or both) significantly decreased decitabine and IR-induced T cell proliferation (Fig. 3b). Thus, these results suggest that decitabine and IR-induced co-stimulatory molecules expression on tumor cells can directly enhance T cell stimulation. Also, we examined whether it can increase T cell-mediated cytotoxicity in the decitabine, IR or both treated tumor cells. A549, HCT-116, and HepG2 cells were treated with 5 μM decitabine for 24 h. Then, tumor cells were exposed to an 8 Gy radiation dose. T cells were co-cultured with the target tumor cells treated by decitabine, IR, or their combination for 4 h; the cytotoxicity assay was performed using flow cytometry. T cell-mediated cytotoxicity against target tumor cells significantly increased by treatment with decitabine and IR alone compared to that in untreated target tumor cells. Furthermore, the combination treatment significantly increased T cell-mediated cytotoxicity compared with either treatment alone (Fig. 3c). Although IR alone increased the cytotoxicity of T cells against target tumor cells than decitabine alone, there was no significant difference. To evaluate the effect of treatment with decitabine and IR-induced MHC class I and co-stimulatory molecules expression in the anti-tumor cytotoxicity of T cells, T cells were co-cultured with target tumor cells in the presence of blocking antibody against MHC class I, CD40 and CD80. As shown in Fig. 3d, blockade of MHC class I, CD40 or CD80 resulted in a substantial reduction of anti-tumor cytotoxicity against target tumor cells, although it was dependent on cell type. In particular, CD80 or MHC class I blockade strongly inhibited the anti-tumor cytotoxicity induced by combination treatment of decitabine with IR against all target tumor cells. Furthermore, the combination blockade of MHC class I and co-stimulatory molecules more significantly reduced T cell-mediated cytotoxicity compared with either blockade alone. These results suggest that decitabine and IR combination treatment further enhances the T cells-mediated cytotoxicity by increasing the expression of MHC class I and co-stimulatory molecules in tumor cells.

### Effects of decitabine and IR combination treatment on IFN-γ production in T cells

**Figure 4:**
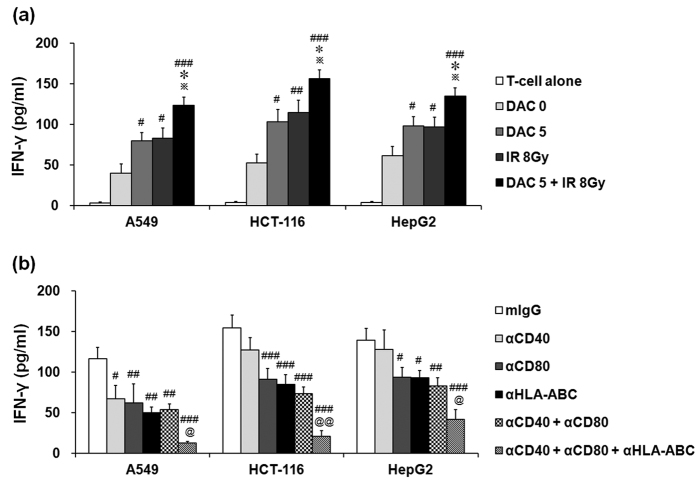
Effects of treatment with decitabine, ionizing radiation (IR), or their combination on IFN-γ production in T cells against target tumor cells. T cells were co-cultured with decitabine, IR or both treated tumor cells (A549, HCT-116, and HepG2 cells) for 4 h (**a**). For blockade experiments (**b**), T cells were co-cultured with combination-treated target tumor cells in the presence of 10 ug/ml anti-CD40, CD80, and/or HLA-A,B,C antibody. Then the cell supernatants were harvested and analyzed by ELISA. Results express the average IFN-γ production ± SD in T cells co-cultured with A549, HCT-116, and HepG2 cells. Experiments were independently performed from five healthy donors. The assay was performed in triplicated each donor. Statistical significance was determined using a one-way ANOVA. ^#^*P* < 0.05, ^##^*P* < 0.005, ^###^*P* < 0.0005 (^#^DAC 0 *versus* other groups, mIgG *versus* other groups). *P < 0.05 (*DAC 5 *versus* other groups). ^**^P < 0.05 (^**^IR 8 Gy *versus* DAC 5 + IR 8 Gy). ^@^*P* < 0.05, ^@@^*P* < 0.005 (^@^αCD40 + αCD80 *versus* αCD40 + αCD80 + αHLA-ABC).

Release of immunomodulatory cytokines such as IFN- γ is one of the major functions of anti-tumor immunity. Tumor cells treated with decitabine and IR alone and in combination were co-cultured with T cells, and IFN-γ levels were subsequently determined in the culture supernatants. Treatment with decitabine and IR alone significantly increased IFN-γ production in T cells compared with untreated target tumor cells; furthermore, the decitabine and IR combination treatment significantly increased IFN-γ levels compared with either treatment alone (Fig. 4a). However, there was no significant difference between treatment with decitabine and IR in the production of IFN-γ. To evaluate the effect of treatment with decitabine and IR-induced MHC class I and co-stimulatory molecules expression in the IFN-γ production of T cells, T cells were co-cultured with target tumor cells in the presence of blocking antibody against MHC class I, CD40 and CD80. As shown in Fig. 4b, blocking of the MHC class I and CD80 resulted in a substantial reduction of IFN-γ production against all target tumor cells, but CD40 blockade significantly reduced only in A549 tumor cells. In particular, the combination blockade of MHC class I and co-stimulatory molecules more significantly reduced T cell-mediated IFN-γ production compared with either blockade alone. These results were similar to those observed in the cytotoxicity test and suggest that treatment with decitabine and IR alone and in combination may further improve the susceptibility of tumor cells to T cell-mediated cytotoxicity.

### Effects of decitabine and IR combination treatment on NKG2D ligand expression in tumor cells

**Figure 5:**
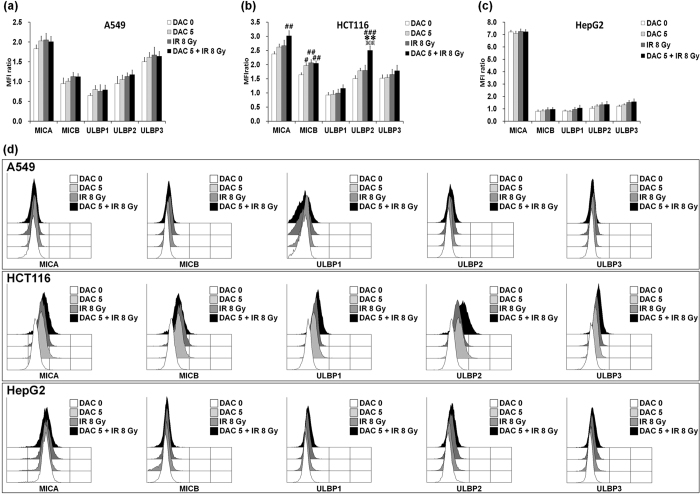
Expression of NKG2D ligands in tumor cells after treatment with decitabine, ionizing radiation (IR), or their combination. NKG2D ligand expression in A549 cells (**a**), HCT-116 cells (**b**), and HepG2 cells (**c**) was analyzed by flow cytometry using specific mAbs. Tumor cells were treated with 5 μM decitabine for 24 h then exposed to radiation doses of 8 Gy. Mean fluorescence intensity (MFI) ratio was calculated as MFI with specific mAb/MFI with isotype control mAb (**a**–**c**). In the figure, filled white represents the untreated control, filled light gray represents decitabine, filled dark gray represents IR and filled black represents decitabine and IR combination treatment (**d**). Results are expressed as the average MFI ± SD. Experiments were independently performed from five healthy donors. The assay was performed in triplicated each donor. Statistical significance was determined using a one-way ANOVA. ^#^*P* < 0.05, ^##^*P* < 0.005, ^###^*P* < 0.0005 (^#^DAC 0 *versus* other groups). **P < 0.005 (*DAC 5 *versus* other groups). ^****^P < 0.005 (^**^IR 8 Gy *versus* DAC 5 + IR 8 Gy).

To investigate whether decitabine and IR combination treatment up-regulates NKG2D ligand expression in tumor cells, A549, HCT-116, and HepG2 cells were treated with 5 μM decitabine for 24 h. Then, the tumor cells were exposed to radiation doses of 8 Gy. NKG2D ligand cell surface expression was quantified using mean fluorescent intensities (MFIs) (Fig. 5). Mean fluorescence intensity (MFI) ratio was calculated as MFI with specific mAb/MFI with isotype control mAb. NKG2D ligand expression was not significantly increased after treatment with decitabine or IR in A549 and HepG2 cells. Also, the combination treatment was negative or only low positive for the NKG2D ligand compared with either treatment alone. HCT-116 cells significantly increased the expression of MICB by treatment with decitabine or IR, but other NKG2D ligands (MICA, ULBP1, ULBP2, and ULBP3) expression did not altered significantly. Although, the combination treatment with decitabine and IR resulted in a significantly increased expression of MICA, MICB, and ULBP2 compared with control cells, it increased only ULBP2 compared with either treatment alone. Previous studies reported that decitabine significantly upregulates NKG2D ligand expression in several cancer cells^12^,^14^,^34^; however, expression levels may differ in individual tumor cells. As such, the results of this study suggest that decitabine treatment does not effectively induce NKG2D ligand expression in A549, HCT-116, and HepG2 cells.

### Effects of decitabine and IR combination treatment on NK cell-mediated cytotoxicity

**Figure 6:**
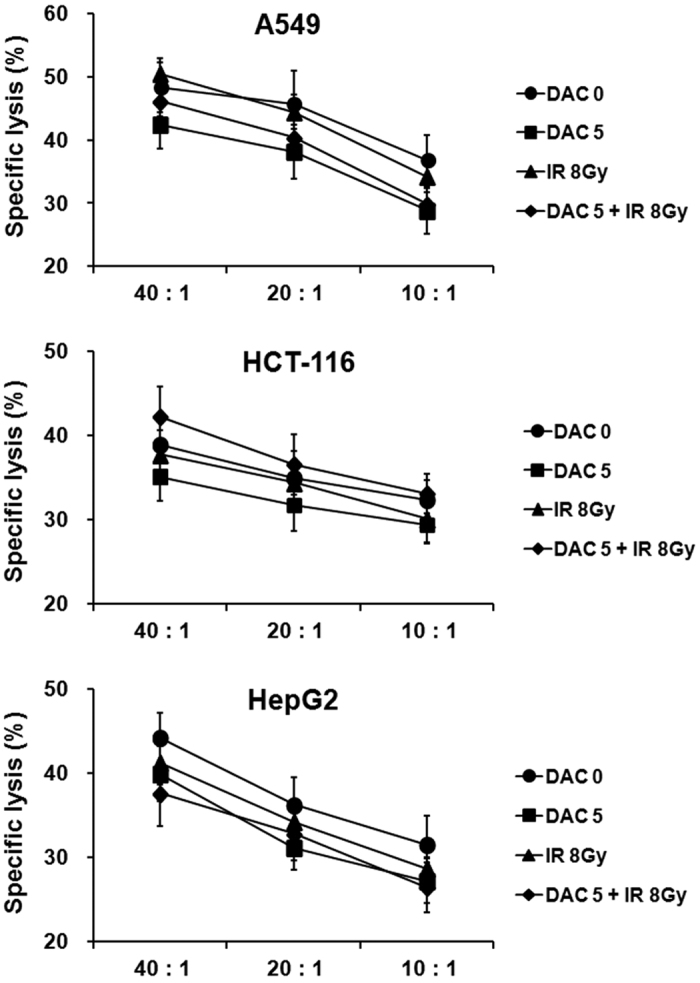
Effects of treatment with decitabine, ionizing radiation (IR), or their combination on cytolytic activity of NK cells against target tumor cells. NK cells were co-cultured with A549, HCT-116, and HepG2 cells at various ratios for 4 h, and cytotoxicity assay was performed by flow cytometry. Results are expressed as average NK cell-induced cytotoxicity ± SD against A549, HCT-116, and HepG2 cells. Experiments were independently performed from five healthy donors. The assay was performed in triplicated each donor. The statistical significance was determined using a one-way ANOVA.

To investigate whether decitabine and IR combination treatment affects NK cell-mediated cytotoxicity in target tumor cells, NK cells were co-cultured with decitabine, IR, or their combination-treated target tumor cells at various ratios for 4 h. Subsequently, a cytotoxicity assay was performed using flow cytometry. NK cell-mediated cytotoxicity against decitabine or IR-treated target tumor cells was slightly decreased in comparison to untreated target tumor cells (Fig. 6); however, the results were not significant. Furthermore, the combination treatment of decitabine and IR also showed a pattern similar to single treatment in NK cell-mediated cytotoxicity. These results may be explained by changes in the expression of MHC class I molecules on target tumor cells observed after treatment with decitabine and IR (Fig. 2a). These results suggest that decitabine, IR, or their combination treatment of target tumor cells does not enhance NK cell-mediated cytotoxicity.

### Effects of decitabine and IR combination treatment on IFN-γ production in NK cells

**Figure 7:**
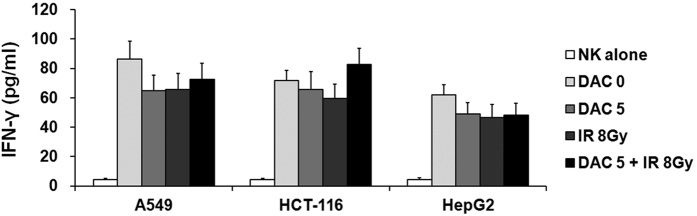
Effects of treatment with decitabine, ionizing radiation (IR), or their combination on IFN-γ production in NK cells against target tumor cells. Tumor cells treated with decitabine and/or IR were co-cultured with NK cells for 4 h. Then the cell supernatants were harvested and analyzed by ELISA. Results express the average IFN-γ production ± SD in NK cells co-cultured with A549, HCT-116, and HepG2 cells. Experiments were independently performed from five healthy donors. The assay was performed in triplicated each donor. Statistical significance was determined using a one-way ANOVA.

Tumor cells treated with decitabine and/or IR were co-cultured with NK cells for 4 h, and IFN-γ levels were subsequently determined in the culture supernatants. As shown in Fig. 7, NK cells alone spontaneously produced very low levels of IFN-γ. Treatment with decitabine or IR did not affect the production of IFN-γ in NK cells compared with untreated target tumor cells. Furthermore, the combination treatment of decitabine and IR also showed a pattern similar to single treatment in IFN-γ production of NK cells. These results suggest that decitabine, IR, or their combination treatment of target tumor cells could not improve the susceptibility of tumor cells to NK cells.

## Discussion

Various genes are abnormally silenced by DNA methylation and these genes are closely associated with immune response and drug sensitivity in cancer cells^3^,^35^. Decitabine (Dacogen^®^) has been used for the treatment of hematological malignancies such as, myelodysplastic syndrome (MDS) and acute myeloid leukemia (AML), and it has been recently considered in the treatment of solid tumors. Treatment with decitabine alone did not show a satisfactory clinical outcome in solid tumors; however, it enhanced the effects of other subsequent treatments such as chemotherapy, immunotherapy, and targeted therapy^36^. Treatment with decitabine up-regulated NKG2D ligand expression in some tumor cell lines^14^; however, this effect was weak when decitabine was used as a monotherapy^13^. In addition, treatment with decitabine decreased NK cell anti-tumor responses in tumor bearing mice^11^.

Immune evasion mechanisms of tumors are a major obstacle to successful tumor immunotherapy^37^. For example, tumor cells can induce mutations or down-regulate MHC class I molecules and tumor antigens^37^,^38^,^39^. These factors suppress the induction of an effective T cell response against tumors. These tumor immunosuppressive factors can be regulated by DNA methylation in tumor cells^5^,^31^,^32^,^40^; therefore, it may be necessary to combine immunotherapy and epigenetic therapy. In this study, we found that treatment with decitabine significantly up-regulated the expression of MHC class I molecules in A549, HCT-116, and HepG2 cells. Furthermore, co-stimulatory molecules such as CD40 and CD80 were significantly increased by decitabine in A549 and HCT-116 cells. However, CD86 expression was not significantly increased in any of the tumor cell lines tested. Importantly, decitabine significantly enhanced the cytolytic activity and IFN-r secretion of T cells in all tumor cell lines tested. These results were more significant differences when combined with irradiation. Treatment with decitabine and IR-induced MHC class I and co-stimulatory molecules strongly induced T cell activation. In contrast, blockade of MHC class I and co-stimulatory molecules significantly inhibited T cell proliferation and cytolytic activity induced by treatment with decitabine and IR. Thus, decitabine may increase the susceptibility of tumor cells to combination T cell-based immunotherapy and radiotherapy. Previous studies reported that epigenetic agents can improve the immunogenicity and susceptibility of tumor cells to immune cells by up-regulating the expression of MHC class I molecules^5^,^40^ and co-stimulatory molecules such as CD40, CD80, and CD86^30^,^32^,^33^. Radiotherapy is one of the most effective treatments for various tumors. Irradiation facilitates cell surface expression of MHC class I molecules^20^,^41^ and CD80^28^,^29^ on tumor cells, and it increases lymphocyte trafficking and sensitizes tumor recognition^20^,^41^. Thus, our results indicate that combination treatment with decitabine and IR are an effective means to enhance immunogenecity and susceptibility of tumor cells to T cells, and these multimodality approaches would be a potential new strategy for T cell-based tumor immunotherapy. In addition, treatment with decitabine in combination with IR may further improve therapeutic effect of radiation by increasing the immune stimulating factors on tumor cells.

NK cells play a crucial role in tumor immune surveillance and are one of the promising therapeutic options for various malignant diseases^16^,^42^,^43^. NK cell activity is regulated by a complex balance between receptors on NK cells and their corresponding ligands on tumor cells^16^,^44^. NKG2D is a key member of activating receptors in NK cells that recognizes MICA/B and ULBPs^15^,^16^. Also, NKG2D ligands show various expression patterns in different tumor cells^45^. In this study, we found that treatment with decitabine and/or IR up-regulated NKG2D ligand expression in HCT-116 cells, but not in A549 and HepG2 cells. Importantly, treatment with decitabine and/or IR slightly decreased cytolytic activity of NK cells in all tumor cell lines tested. These results may be due to a significant increase in MHC class I molecules on tumor cells treated with decitabine and/or IR. Previous studies with several solid tumor and AML cells have shown that treatment with decitabine could up-regulate NKG2D ligand expression which contributes to increased NK cell-mediated cytolytic activity^12^,^13^,^14^,^34^; however, this effect was weak when decitabine was used as a monotherapy^13^. Tang *et al*. reported that treatment with decitabine induced MICB expression in HepG2 and HEK293T cells and sensitized cells to NK cell-mediated cytolysis^14^. Here, our results indicated that this effect was not clear in A549 and HepG2 cells. These cells showed only very low expression level for the different NKG2D ligands. However, HCT-116 cells were increased the expression of MICB by treatment with decitabine. The different observations may be due to different tumor cells characteristics, different drug treatment periods, and different NK cell preparation. In contrast, decitabine increases KIR expression on NK cells, consequently leading to decreased NK cell-mediated cytolytic activity *in vitro*^11^,^12^,^27^. In addition, treatment with decitabine decreased the number and activity of NK cells in tumor bearing mice^11^.

The NK cell inhibitory signal is regulated through KIRs binding to MHC class I molecules expressed on tumor cells. Thus, tumor cells with high expression of MHC class I molecules are resistant to NK cells^15^,^16^,^46^. Decitabine did not effectively induce the expression of NKG2D ligand in tumor cells, and combination treatment strongly up-regulated MHC class I molecules in tumor cells (Fig. 4). These results suggest that the combination treatment of decitabine with IR could down-regulate NK cell-mediated cytotoxicity.

Taken together, we found that decitabine impairs the cytolytic activity of NK cells by up-regulating the expression of MHC class I molecules in tumor cells, while strongly enhancing T cell effector functions by up-regulating the expression of MHC class I as well as its co-stimulatory molecules in tumor cells. These results suggest that decitabine may be more effective when combined with other tumor therapies such as conventional therapy, immunotherapy (particularly T cells and dendritic cells), and targeted therapy. Thus, our results indicate that decitabine may be a useful immunomodulator for tumor cell sensitization.

## Methods

### Ethics statement

All the methods were carried out in accordance with the approved guidelines at Dongnam Institute of Radiological & Medical Sciences. All experiments using human blood were approved by the Ethical Committee of Dongnam Institute of Radiological & Medical Sciences (D-1508-002-001), and written informed consent was obtained from all the donors before enrollment.

### Cell culture

The human lung cancer cell line A549, colon cancer cell line HCT-116, and hepatoma cancer cell line HepG2 were purchased from the American type culture collection (Rockville, MD, USA). Cells were cultured in RPMI 1640 (WELGENE, Seoul, Korea), McCoy’s 5A (WELGENE), and DMEM (WELGENE) supplemented with 10% (v/v) fetal bovine serum (FBS, GIBCO BRL, Grand Island, NY, USA), 1 mM L-glutamine, 100 U/ml penicillin, and 100 mg/ml streptomycin at 37 °C in a humidified atmosphere of 95% air and 5% CO_2_. Decitabine was purchased from Sigma-Aldrich Chemical Co. (St. Louis, MO, USA), and dissolved in 100% dimethyl sulfoxide (DMSO, Sigma-Aldrich) to obtain a stock concentration of 10 mM and then stored at −80 °C.

### MTT assay

Cells were seeded onto 6-well plates at a concentration of 5 × 10^4 ^cells/well, grown to 70% confluence, and then treated with decitabine (0, 2.5, 5, and 10 μM) for 24 h. Following treatment, cell viability was determined using the 3-(4,5-dimethylthiazol-2-yl)-2,5-diphenyl-tetrazolium bromide (MTT, Sigma-Aldrich) assay, which is based on the conversion of MTT to MTT-formazan by mitochondrial enzymes.

### IR exposure

Tumor cells treated with decitabine (0, 5 uM) for 24 h and untreated tumor cells applied at a dose of 8 Gy using a linear accelerator (Infinity; ELEKTA, UK). Following irradiation, cells were incubated for 24 h at 37 °C in a humidified incubator with 5% CO_2_.

### Flow cytometry

A549, HCT-116, and HepG2 cells were seeded in 60 mm plates for 24 h and then treated with a predetermined decitabine dose for 24 h at 37 °C in a humidified incubator with 5% CO_2_. Cells were harvested by trypsinization and washed twice with cold PBS. They were then immunostained with anti-mouse MICA, MICB, ULBP1, ULBP 2/5/6 and ULBP3 phycoerythrin (PE) (R&D Systems, Minneapolis, MN, USA), and anti-mouse HLA-A,B,C fluorescein isothiocyanate (FITC) (Beckman Coulter, CA, USA) at 4 °C for 30 minutes. Flow cytometric analysis was performed with a FC500 flow cytometer (Beckman Coulter).

### Mixed Lymphocyte Reaction (MLR)

T cells isolated from 5 healthy donors were used as responder cells. Decitabine, IR, their combination, and vehicle-treated A549, HCT-116, and HepG2 cells fixed with 4% paraformaldehyde were used as stimulator cells. Responder cells and stimulator cells were incubated in 96-well plates in a ratio of 5:1, and cultured at 37 °C at 5% CO_2_ for 5 days. T-cell proliferation assays were assessed with Cell Counting Kit-8 (Dojindo, Kumomoto, Japan) according to the manufacturer’s instructions. Briefly, after incubation for 5 days, 10 μl tetrazolium salt (WST)-8 was added to each well, and the cells were incubated for 4 hours at 37 °C. The absorbance of the sample at 450 nm was measured. For co-stimulatory molecules blockade, anti-CD40 (clone 5C3) and CD80 (clone 2D10) or a control isotype matched IgG, were added into co-culture wells at a final concentration of 10 μg/ml. All antibodies were purchased from BioLegend (San Diego, CA).

### T cell and NK cell cultures

Peripheral blood mononuclear cells (PBMCs) were isolated from 5 healthy donors using Histopaque-1077 (Sigma-Aldrich) density gradient centrifugation. For T cell expansion, PBMCs were re-suspended at 1 × 10^6 ^cells/ml in lymphocyte growth medium 3 (LGM-3) (Lonza, Basel, Switzerland) containing 10 ng/ml anti-CD3 antibody (OKT3, eBioscience, San Diego, CA, USA), 500 IU/ml recombinant human interleukin-2 (rhIL-2 (Proleukin^®^), Novartis Pharmaceuticals Corp., NJ, USA), and 5% human serum (Sigma-Aldrich). On day 5, expanded T cells were transferred to a larger culture flask in LGM-3 medium containing 500 IU/ml rhIL-2 and 5% human serum. Fresh culture medium containing rhIL-2 (500 IU/ml) and 5% human serum was added to the flask every 2 to 3 days for 14 days. Purified NK cells were obtained by depleting non-NK cells with a magnetic activated cell sorting (MACS) system according to the manufacturer’s instructions (Miltenyi Biotec, Bisley, Germany). NK cell purity was assessed by flow cytometry using anti-human CD3 and CD56 mAbs. Purified NK cells were cultured for 7 days in LGM-3 containing rhIL-2 (500 IU/ml) and 5% human serum. All procedures were performed under conditions of good manufacturing practices (GMP).

### Cytotoxicity assay using flow cytometry

A549, HCT-116, and MCF-7 cells (target cells) were labeled with carboxyfluorescein succinimidyl ester (CFSE, Sigma-Aldrich) at a final concentration of 5 μM for 15 min at 37 °C in a humidified incubator with 5% CO_2_. After labeling, cells were washed with complete medium. Effector cells were co-cultured with each CFSE-labeled cell line at the appropriate effector-to-target cell count ratios (40:1, 20:1, and 10:1) in round-bottomed 96-well plates at 37 °C in a humidified incubator with 5% CO_2_ for 4 h. After incubation, the cells were transferred to tubes and placed in an ice water bath. Propidium iodide (PI, Sigma-Aldrich) (50 μg/ml) was added to label the DNA of dead cells and then analyzed by flow cytometry (Beckman Coulter, CA, USA). For co-stimulatory molecules (CD40 and CD80) and MHC class I blockade experiments, T cells were co-cultured with combination-treated tumor cells in the presence of 10 ug/ml anti-CD40, CD80, or HLA-A,B,C (clone W6/32, BioLegend) antibody.

### IFN-γ enzyme-linked immunosorbent assay (ELISA)

Effector cells were co-cultured with each tumor cell line at the effector-to-target cell count ratio (10:1) in round-bottomed 96-well plates at 37 °C in a humidified incubator with 5% CO_2_ for 4 h. Each sample was then centrifuged at 1,500 rpm for 5 min at room temperature and the supernatant (co-culture medium) was collected and stored at −80 °C until analysis. IFN-γ levels in the supernatant were determined using commercially available ELISA kits (Mabtech, Nacka Strand, Sweden) according to the manufacturer’s instructions. Results are reported as pg/ml total protein. For co-stimulatory molecules (CD40 and CD80) and MHC class I blockade experiments, T cells were co-cultured with combination-treated tumor cells in the presence of 10 ug/ml anti-CD40, CD80, or HLA-A,B,C antibody.

### Statistical analysis

Statistical analysis was performed using one-way analysis of variance (ANOVA) and paired Student’s t-test. Differences were considered statistically significant at *P* < 0.05.

## Additional Information

**How to cite this article**: Son, C.-H. *et al*. Combination treatment with decitabine and ionizing radiation enhances tumor cells susceptibility of T cells. *Sci. Rep.* **6**, 32470; doi: 10.1038/srep32470 (2016).
